# Effect on osseointegration of two implant macro-designs:A histomorphometric analysis of bicortically installed implants in different topographic sites of rabbit’s tibiae

**DOI:** 10.4317/medoral.22825

**Published:** 2019-06-25

**Authors:** David Soto-Peñaloza, Marco Caneva, José Viña-Almunia, José-Javier Martin-de-Llano, Berta García-Mira, David Peñarrocha-Oltra, Daniele Botticelli, Miguel Peñarrocha-Diago

**Affiliations:** 1 Oral Surgery and Implantology Division, Stomatology Department, University of Valencia, Valencia, Spain; 2 ARDEC Academy, Ariminum Odontologica, Rimini, Italy; 3 Department of Pathology and Health Research Institute of the Hospital Clínico (INCLIVA), Faculty of Medicine and Dentistry, University of Valencia, Valencia, Spain

## Abstract

**Background:**

To evaluate the effect of two different implant macro-designs on the sequential osseointegration at bicortically installed implants in the rabbit tibia. A further aim is to compare the osseointegration at different topographic zones.

**Material and Methods:**

27 New Zealand rabbits were implemented. Two implants, one for each macro-design (Ticare Inhex® or Ticare Quattro®, Mozo-Grau, Valladolid, Spain), were randomly implanted in the diaphysis or metaphysis of each tibia. The flaps were sutured to allow a submerged healing. The animals were sacrificed after 2, 4 or 8 weeks. Ground sections were prepared and analyzed.

**Results:**

No statistically significant differences were found between the two groups for newly formed bone in contact with the implant surface, being about 16%, 19% and 33% in both groups, after 2, 4, and 8 weeks of healing. Bone apposition was slightly higher in the diaphysis, reaching values of 36.4% in the diaphysis, and 29.3% in the metaphysis at 8 weeks of healing. It was observed that the implant position showed a statistical significance regarding BIC values at 4 and 8 weeks (*p*<0.05). Multivariate analysis fails to detect statistical significant differences for the interaction between implant designs and topographic site. Ticare Quattro® design had a slight better BIC values at diaphysis sites across healing stages, but without reaching a statistical significance.

**Conclusions:**

The both implant macro-designs provided similar degrees of osseointegration. Bone morphometry and density may affect bone apposition onto the implant surface. The apposition rates were slightly better in diaphysis compared to metaphysis.

** Key words:**Animal study, bicortical stabilization, implant macro-design, osseointegration, dental implant, submerged healing.

## Introduction

Osseointegration and the direct bone-to-implant contact (BIC) (1) are concepts that transformed the maxillofacial reconstruction approaches. Among factors that may exert an effect on bone to implant interfacial remodeling and new bone apposition, the implant material, the surgical technique, the host bed, the implant design and surface, the time and loading conditions showed to affect osseointegration (2,3). To achieve implant integration, the primary stability is a key goal required to avoid fibrous encapsulation (4).

Primary stability is the mechanical interlocking between the implant and the surrounding bone, which is influenced by the implant macro-geometry, surface roughness and surgical preparation (5). Further, this primary stability decreases when a remodeling of the surrounding parent bone takes place. It is responsible of the implant stability dip, that simultaneously concurs with a secondary or biologic stability gain “osteoconduction” which depends in great extent of the implant surface roughness, and its capacity on fibrin clot retention during healing (6-8).

It is suggested that bone remodeling occurs depending on the degree of mechanical stress (9); So it of utmost importance that the thread design provides a certain level of static strain to the surrounding bone (10). Threads are also used to maximize initial contact, improving initial stability and enlarging the implant surface area (11), that favors dissipation of interfacial stress (12). Different implant thread designs and thread pitches were proposed aiming to enhance and optimize the osseointegration process, as well as in loading conditions (13). Implant geometry was reported to affect the BIC ratio and mechanical test values (14). Also, modified macro-geometry and different microgeometries of implants has shown to have an stimulatory effect on osseointegration (10), that impacts the dynamics of implant osseointegration and suggesting that macro-design features should be made relative to the biological and mechanical micro-environment (15).

Nevertheless, other characteristics such healing chamber configuration have proven to facilitate osseointegration (16). Pre-clinical evidence on removal torque values (17), and osseointegration at different titanium surfaces (18), or at implants with different apical configuration design (19) are available for bicortically placed dental implants. However, data on bicortically installed implants with different macro-designs are still missing. Hence, the aim of the present experiment was to evaluate the effect of two different implant macro-designs but equal surface roughness on the sequential osseointegration at bicortically installed implants in the rabbit tibia.

## Material and Methods

This animal study was performed in accordance with the ARRIVE guidelines (20). The relevancy of the animal selection and its use were carefully established and considered.

-Ethical statement

The study protocol was submitted to and approved by the Ethics Committee of Valencia University, Spain (Protocol ref.: A1432625410189), which followed the guidelines established by the Council Directive of the European Union (53/2013; February 1, 2013) for animal care and experimentation in agreement with the ethical and legal conditions established by Royal Decree 223, March 14 and October 13, 1988.

-Study design and experimental animals

The present experimental pre-clinical study involved twenty-seven males, albino New Zealand rabbits (Rabbit Farm San Bernardo, SL, Navarra, Spain) with general register of livestock farms (REGA) code: ES312330000101, the mean age was 24 weeks and the weighing around 3 to 4 kg. The animals were divided into three groups composed of 9 animals each and sacrificed at 2, 4 and 8 weeks, respectively. Each animal received randomly four dental implants, two each tibiae (diaphysis and metaphysis).

-Randomization and allocation concealment

The animals were randomly assigned to one of the three groups, each group was the representation of each one of the healing periods. Two implants with a different macro-design were installed in each tibia. The position of each implant, i.e. diaphysis or metaphysis, was randomly assigned. The randomization was carried out electronically (www.randomization.com) by an independent author involved neither in the selection of the animals nor in the surgical procedures (DB).

-Implant macro-design features

**Figure 1:**
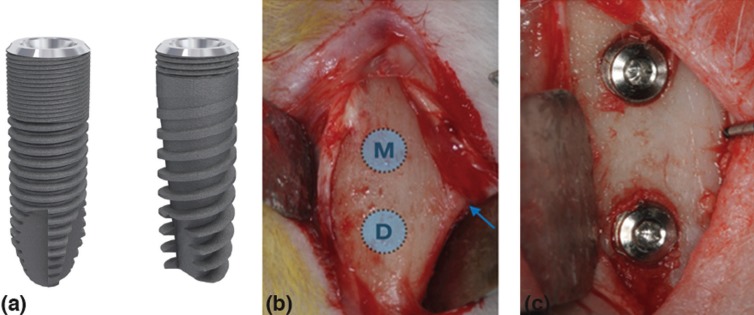
(a) Image of different implant macro-designs tested. Inhex® (Left) and Quattro® (Right) (Ticare implants, Mozo-Grau, Valladolid, Spain). Ticare Inhex®: the implant body had a little conicity and a large area of micro-threads at the coronal portion, and higher number of triangular threads per unit length and with little thread depth compared to Quattro® model. Moreover, the implant had a double self-tapping at the apical portion. Ticare Quattro®: the implant body had a marked conicity. Fewer micro-threads at the coronal portion and a lower number of macro-threads were present compared to Inhex implants. The threads were squared in the middle part of the implant and become triangular and deeper at the apex. Aggressive self-tapping at the apex. (b) The flaps were raised, and the bone was exposed below the anterior tibial tuberosity (blue arrow), that provides a visual reference point to identify the two experimental sites, one in metaphysis “M” and one in the diaphysis “D”. Thereafter, two implants macro-designs were bicortically installed in each tibia (c).

Ticare® implants (Mozo-Grau, Valladolid, Spain) made of commercially pure grade-IV titanium treated with resorbable blast media (RBM) surface (implant surface blasted with calcium phosphate ceramics) were used. All implants had a dimension of 3.75 mm of diameter and 8 mm of length, a conical connection with a 45º polish platform with a self-tapping feature closer to the apex. Two different macro-designs based on thread type were tested (Fig. 1a,b).

-Clinical procedures

The rabbits were anesthetized with intramuscular injection of Ketamine (22mg/kg) and xylazine (2.5 mg/kg) were administered at 50% and intravenous injection of Propofol (1.5mg/kg) and maintained with 2% of isofluorane. Before surgery, the skin at the proximal tibia was shaved and disinfected with Betadine. A preoperative antibiotic Enrofloxacin 5mg/Kg (ALSIR® 2,5%, Esteve Veterinaria, Barcelona, Spain) was administered subcutaneously, and 3 ml of articaine at 2% with 0.01 mg/ml epinephrine infiltrative anesthesia was also administered intramuscularly in the surgical area of each leg. The skin of both tibiae was incised in the proximal region (Fig. 1c). Two experimental sites were identified in each tibia (Fig. 1d). The recipient sites were prepared using drills with increasing diameter under irrigation with sterile saline according manufacturer. A distance of about 8-10 mm was maintained between the two osteotomies. Two implants with different macro-design were randomly installed in each tibia, and were screwed until the implant shoulder was leveled with the bone surface. The apex of the implants was placed in close contact with or into the cortical bone opposing the coronal cortical compartment, aiming to obtain a bicortical anchorage. The cover screws were placed on the implants, and the flaps were subsequently sutured in layers with resorbable sutures to allow a submerged healing (Vicryl 5/0, Ethicon, Sommerville, NJ, USA), and Nylon 3/0 (Ethilon 3/0, Ethicon, Sommerville, NJ, USA).

-Pre- and Post-operative care, housing and husbandry

All animals were kept in individual cages during its acclimatization period before intervention (2 weeks) and during post-operative care at the Animal Room Service Unit, University of Valencia, Spain, in purpose-designed and acclimatized rooms at 21ºC with 12 h dark/light ambiance. The animals were fed with a standard diet and had free access to water. The analgesic pattern consisted in 2.5mg/kg of morphine intraoperative, 0.02 mg/kg buprenodale, buprex, 0.2 mg/kg meloxicam (every 12 hours during 3 days) and antibiotic therapy with Enrofloxacin 2.5 mg/Kg (ALSIR® 2,5%, Esteve Veterinaria, Barcelona, Spain) (every 24 hours during 7 days) post-operatively.

-Euthanasia

Nine rabbits of each three groups were euthanized after 2, 4 and 8 weeks, respectively. The same sedation and anesthesia protocols, such as for the surgery, were applied and the euthanasia induction was performed with 50mg/kg intravenous sodium pentobarbital. A small electric saw was used to obtain the sections of the tibia containing each implant.

-Histological preparation

Implant samples were dehydrated by sequential solvent exchange and embedded in methyl methacrylate containing poly-(methyl methacrylate). After adding benzoyl peroxide (1 g/100 mL), samples were polymerized and were then sawed using a diamond wheel on a precision table top cut-off machine Accutom-5, (Struers, Copenhagen, Denmark) and then were wet ground and polished using a LaboPol-21 system (Struers, Copenhagen, Denmark) and SiC foils. Approximately 80 μm thin sections were obtained. The samples were stained at 55ºC with toluidine blue for 30 min, washed with tap water for 2 minutes and let dry.

-Histological examination

Overlapping calibrated digital images of the tissues surrounding the whole implant surface (about 20 images/implant) were recorded with a bright field Leica DM4000 B microscope (Leica Microsystems GmbH, Wëtzlar, Germany) and DFC420 digital camera using a 5× objective and the Leica Applications Suite version 4.4.0 software. Individual images were merged to compose each implant side using the Photoshop program (Adobe Photoshop CC 2015.0.0, Adobe Systems Incorporated, San José, CA, USA, http://www.adobe.com/Photoshop). The image processing program ImageJ 1.48 (National Institutes of Health, Bethesda, MD, USA; http://imagej.nih.gov/ij) was used for histological measurements. Lines were drawn by hand on calibrated images showed on the computer screen at a 400× magnification by an independent and calibrated assessor not involved in study. The landmarks identified are the same as described previously by our group (21).

The BIC was evaluated as the sum of new and old bone, and percentages in relation to the length of the implant surface examined calculated. The apical portion of the implant that extruded beyond the compact cortical layer was excluded from the analyses.

-Data analysis

Differences between implant designs across the healing periods were analyzed with the Mann–Whitney U-test for independent variables. Differences between implants placed in the diaphysis and metaphysis were also performed using a Wilcoxon rank-sum test. A multivariate general lineal model analysis was performed to explore the interaction between the two independent variables (design/position) over BIC values at different healing stages. Each factor with two categories: design (Ticare Inhex®/Ticare Quattro®) and position (diaphysis/metaphysis). This approach was chosen because previous reports observed that the positions of the implants can be used as independent replicates regarding outcome variable, since bone quality varies between implantation sites (topographic sites) at same degree as between experimental units (22).

## Results

-Clinical and histological outcomes

No complications occurred during the healing period. All implants seemed adequately integrated into the histological evaluation across each period. Finally, data of 27 experimental animals with four implants each were analyzed. The areas between the threads were filled with woven bone at two weeks. Remodeling processes were observed after 4 and 8 weeks of healing, as shown by the lighter-staining of the lamellar bone compared to the darker-staining of the woven bone. The summary of results at 2, 4 and 8 weeks, for both implant design and topographic location (diaphysis or metaphysis) are depicted in  Table 1 and  Table 2.

**Table 1:**
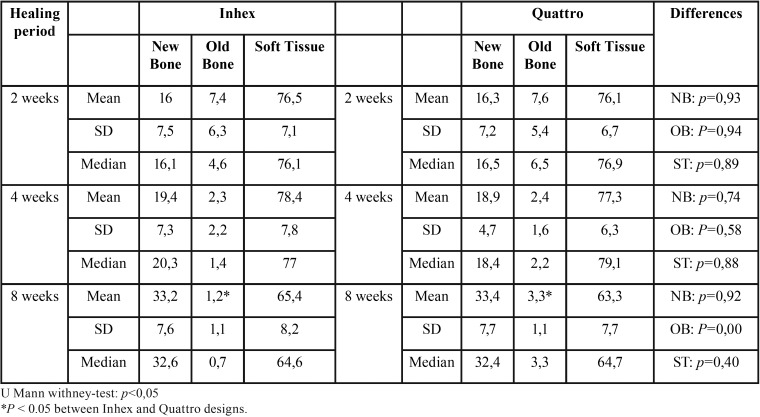
Summary of proportion (%) of tissues components according implant macro-designs, (n = 9) per each period of healing.

**Table 2:**
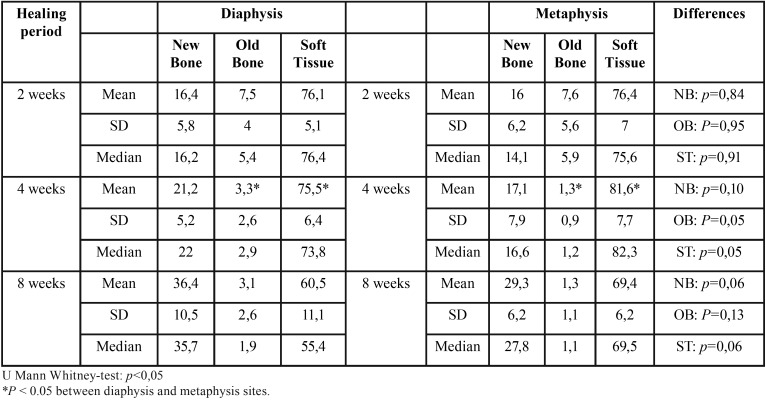
Summary of proportion (%) of tissues components according implantation site, (n = 9) per each period of healing.

-2-week healing

**Figure 2:**
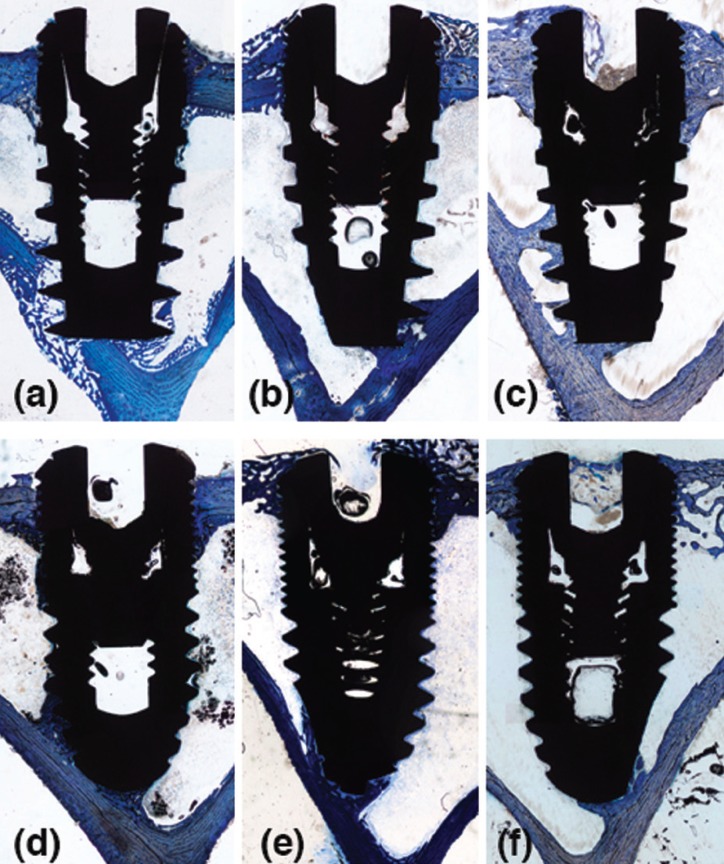
Ground sections illustrating the healing of implants installed in the diaphysis (a-c) and metaphysis (d-f) areas after 2, 4 and 8 weeks. Toluidine blue (1.6x).

**Figure 3:**
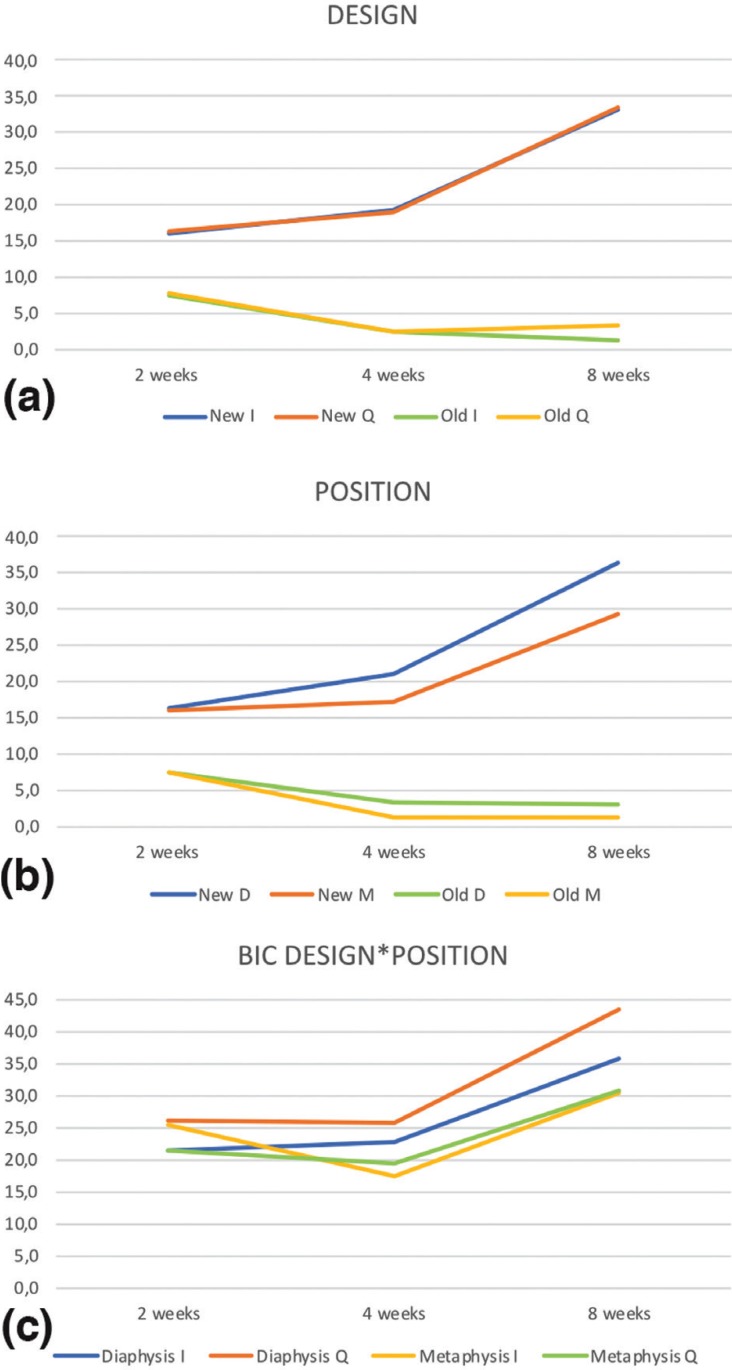
(a) Graphics reporting the amount of new bone and old bone for Inhex® (I) and Quattro® (Q) implant designs (New I; New Q) and (Old I; Old Q) at different time intervals respectively. (b) Differences of new bone (New D; New M) and old bone (Old D; Old M) at diaphysis (D) or metaphysis (M) sites. (c) BIC values for both macro-designs according implantation site (diaphysis or metaphysis), to visually appreciate the interaction (Design*Position).

Ground sections illustrating the healing after 2 weeks are presented in Figure 2; for the diaphysis (Fig. 2a) and metaphysis zones (Fig. 2d). A similar degree of new osseointegration was observed in both macro-designs at this stage, being 16.0±7.5% for Ticare Inhex®, and 16.3±7.2% for Quattro® implants. The old bone percentages observed were around 7.4% and 7.6% for Ticare Inhex® and Quattro® implants, respectively (Fig. 3a). Regarding implant position, there were not significant differences among the assessed parameters at this stage (Fig. 3b). Similar BIC% were observed between implant macro-designs and regarding topographic site placement being 23.5±14.4% and 23.9±13.3 % for Ticare Inhex® and Quattro® implant designs, respectively. None of the differences for both macro-design and topographic sites was statistically significant ( Table 1, Table 2).

-4-week healing

Ground sections illustrating the healing after 30 days are presented in for the diaphysis (Fig. 2b) and metaphysis (Fig. 2e) zones, respectively. The values of new osseointegration at this time of healing were 19.4±7.3% and 18.9±4.7% for the Ticare Inhex® and Quattro® designs, respectively. Old bone percentages at this stage were 2.3±2.2% and 2.4±1.6%, respectively ( Table 1; Fig. 3a). Grouping the data according the implant position in the diaphysis and metaphysis, there was not significant difference for new osseointegration (*p*=0.10). However, a significant difference found for old bone and soft tissue values at this stage ( Table 2; Fig 3b). Similar BIC values (old + new bone) were observed between implant macro-designs, but regarding topographic site placement better BIC values for diaphysis 24,5±6,2% than metaphysis 18,4±7,7 % at this stage (*p*=0,05).

-8-week healing

At this stage, new bone increased, reaching percentages of 33.2±7.6% and 33.4±7.7% for Ticare Inhex® and Quattro® implant designs, respectively ( Table 1; Fig. 3a). No statistically significant differences were found between the two groups. Old bone was still present, however at very low percentages, being 1.2±1.1% and 3.3±1.1% for Ticare Inhex® and Quattro® designs, respectively (*p*=0.001). The new bone percentages in the diaphysis was 36.4±10.5% while in the metaphysis was 29.3±6.2% ( Table 2; Fig 3b). No statistically significant differences were found. The BIC values observed between implant macro-designs do not showed a significant difference, even though slight better BIC values in favor Ticare Quattro® compared to Inhex® design were found, being 36.7±7.7 % and 34.4±7.8, respectively. However, regarding the topographic site placement, a better BIC value for diaphysis (39.5±11.1%) than metaphysis sites (30.6±6.2%; *p*=0.05) was seen at this stage of healing.

-Multivariate analysis

It was observed that the implant position showed a statistical significance regarding BIC values at 4 and 8 weeks (*p*<0.05). However, the analysis fails to detect statistical significance for implant macro-designs and its interaction (design*position) over BIC values across healing stages. Descriptive data is summarized in  Table 3. A visual interaction is appreciated suggesting that the position affects osseointegration values (Fig. 3c). Also, is observed that Ticare Quattro® design showed a slight better BIC values at diaphysis sites across healing stages (*p*>0.05).

**Table 3:**
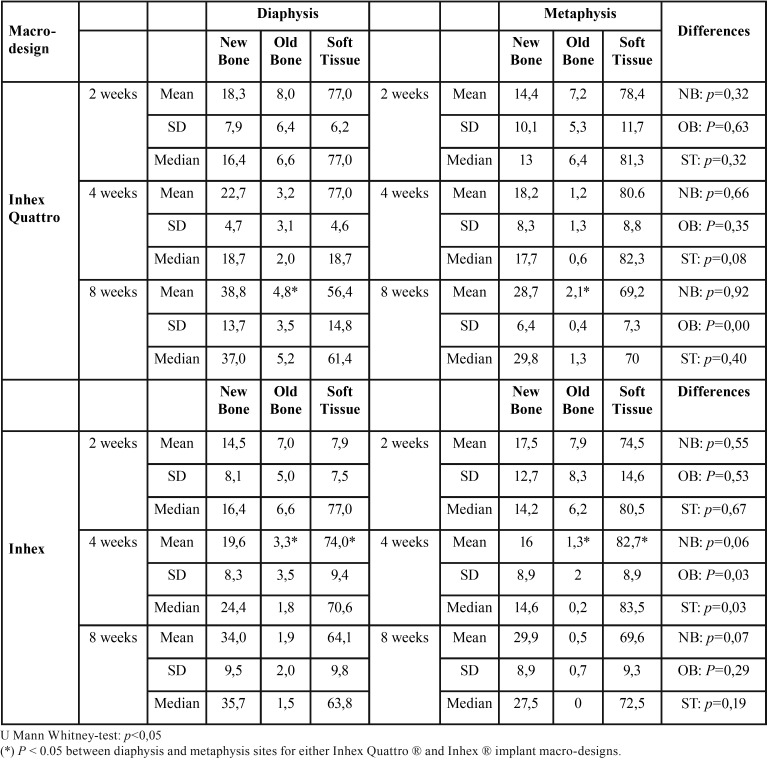
Summary of proportion (%) of tissues components according implant macro-designs regarding topographic site, (n = 9) per each period of healing.

## Discussion

The present study is focused on the bone response over two different implant macro-designs with equal RBM surface treatment and the same length and diameter, bicortically installed in the tibia of the rabbit. The study was performed with the aim of assessing the influence of macro-geometry on osseointegration. To isolate the possible effect of implant macro-geometry on bone formation, both implants had the same surface treatment. In order to appreciate the behavior of both implant macro-designs in two different bone environment, they were placed in two topographic zones within the same tibia, one with a cortical layer and a medullar content (diaphysis) like a type II bone and the another more trabecular like a type III bone (metaphysis). The histomorphometric analysis at either 2, 4 and 8 weeks were similar (*P* > 0.05) for both implant designs.

Moreover, comparing new bone percentages in relation to the topographic implant placement, after 4 and 8 weeks of healing, osseointegration was found to be slightly higher, but statistically not significant at the implants placed in the diaphysis compared to the metaphysis. These findings are contrary to those reported in a previous experiment in rabbits (18). Observations that could be attributable to several factors, such as the implant thread design, the surface treatment tested and the implant osteotomy protocols, differing between studies. It is known that these factors could regulate the strain applied to hard tissue in proximity to the implant (23).

Old bone was resorbed, but was still present after 1 month of healing (<4%), with statistical significant better values in Quattro® group. This pattern of healing is in agreement with other studies performed in animals (8,18,24) and humans (25). Noteworthy to mention, bone morphology in diaphysis is predominantly occupied by a marrow content in comparison to metaphysis that presents more trabecular bone. These findings are in agreement with the assumption that osseointegration is faster in zones where the bone apposition is not preceded by bone resorption (8). It appears likely that bone formation started from the cortical compartments (in contact with mineralized parent bone) and, subsequently, proliferated toward into the marrow compartments. The implants were in close contact to pristine bone due to its bicortically stabilization, a condition that favors osseointegration on the implant surface. A pattern of healing that were documented for osseointegration in different pre-clinical models (26-28).

The parent old bone in recipient site is responsible of mechanical interlocking, and thereafter it is relevant during implant stability dip, where takes place a cell mediated interfacial bone remodeling (6). This is typically described to occur in the area of contact between the pristine bone wall and implant surface, where remodeling arise in the proximity of microcracks followed by bone apposition in void spaces resulting in secondary stability (29). The results from the present study are in agreement with other studies that showed that macro-design did not significantly affect the BIC rates under the absence of loading conditions (30). However, the above report not differentiate the discrepancies regarding implant positioning within rabbit tibiae in its analysis, a factor that may probably contribute to results, due to the different bone density between more caudal to more cranial positions within the tibia. Also a previous report suggests that implant macro-design features, such thread pattern and thread pitch, can be responsible for differences in the amount of bone and degree of apposition toward the implant surface.

Therefore, consideration of specific implant macro-design should be made relative to the biological and mechanical microenvironment (15). However, due to the absence of functional load, these parameters reflect the structural connection between implant and bone, and not the functional properties of the bone to implant interface (30). There is scarce pre-clinical evidence regarding sequential healing of bicortically installed implants with two macro-designs and equal surface treatment, attempting to assess its interaction in two topographic sites. Despite that the interaction of factors was assessed, only the topographic site seems to contribute to values at 4 and 8 weeks. Regrettably, the test not detect significant differences, it is owing the scarce sample for this comparison, that conditionate a lack statistical power in this analysis. So, further studies are warranted, with a greater sample for this aspect, but stressed challenge considering ethical and economical aspects that may involve.

## Conclusions

Despite the limitations of this study due to its pre-clinical nature as well as the aspects above mentioned, we may conclude that both implant macro-designs provided similar degrees of osseointegration. Bone morphometry and density may affect the bone apposition onto the implant surface. The apposition rates were slightly better in diaphysis compared to metaphysis.
